# Hepatocellular Carcinoma Recurrence in HCV Patients Treated with Direct Antiviral Agents

**DOI:** 10.3390/v11050406

**Published:** 2019-05-01

**Authors:** Marco Sanduzzi-Zamparelli, Loreto Boix, Cassia Leal, María Reig

**Affiliations:** 1 Barcelona Clinic Liver Cancer (BCLC) Group, Liver Unit, Hospital Clínic Barcelona, IDIBAPS, University of Barcelona, 08036 Barcelona, Spain; MSANDUZZI@clinic.cat (M.S.-Z.); LBOIX@clinic.cat (L.B.); GUEDES@clinic.cat (C.L.); 2 Centro de Investigación Médica en Red de Enfermedades Hepáticas y Digestivas (CIBERehd), Instituto de Salud Carlos III, 28029 Madrid, Spain

**Keywords:** hepatitis C virus, hepatocellular carcinoma, liver cancer, recurrence, sustained virologic response, DAA

## Abstract

The risk of hepatocellular carcinoma recurrence is universal regardless of the treatment modality applied, and secondary prevention is still an unmet issue even though the elimination of hepatitis C (HCV) with direct antiviral agents (DAAs) was expected to be one of the new options. Unfortunately, the impact of DAAs on hepatocellular carcinoma (HCC) development (de novo and recurrence) is still controversial. Since the first publication on the subject in 2016, almost all groups worldwide have carried out research in this field with hundreds of publications now available. This revision is focused on the impact of DAAs on HCC recurrence and aims to discuss the potential underlying mechanisms and host factors pointing out the time association phenomenon between DAA treatment and HCC recurrence. Moreover, we comment on the methodological issues that could affect the different interpretations of the published results. In conclusion, this is an area of research with potential in the understanding of the impact of factors not previously considered, and may also help change hepatocarcinogenesis tenets, such as the belief that the elimination of HCV should be used as a second prevention treatment.

## 1. Introduction

The challenge of curing hepatitis C (HCV) has already been met. Over 100 million patients, the majority with liver cirrhosis, have been cured (data of 2015) [1]. Despite improved liver function in almost all patients and a decrease in cirrhosis complications, the natural history of these cured HCV patients is unknown. The scientific community currently has the challenge of understanding why some individuals do not modify their own risk of developing liver cancer (LC)—incidence or recurrence—despite having been cured of HCV. The debate regarding the impact of direct antiviral agents (DAAs) on LC development is still open despite the fact that part of the scientific community considers that this issue is merely a reflection of a biased interpretation of the data. The controversy is reflected in some recommendations, such as those that suggest delaying DAA initiation from 6 to 12 months in patients with prior history of hepatocellular carcinoma (HCC) to decrease the risk of HCC recurrence. If there is no change in the recurrence rate post-DAA treatment, why should we wait to treat the patients? Since the first manuscript published in the field, more than 600 manuscripts [2] have focused on this topic but only a few of them have evaluated the mechanisms related to this phenomenon. Thus, the aim of this review is to summarize the mechanisms or host factors that could impact LC development in the setting of DAA treatment or after ending exposure to DAAs.

Issues such as the impact of liver function, the already known influence of metabolic factors or a discussion on the increase/decrease of de novo incidence and/or HCC recurrence rate have not been covered here as these have already been covered in several revisions within the last year [3,4,5,6,7,8,9].

## 2. Pathogenesis of HCC Development after DAA Therapy

In this regard, several topics should be considered in the effort to fully understand the pathogenesis of HCC development after DAA therapy. First of all, the appearance of DAA-resistant HCV strains should be taken into account [10]. The presence of active strains of HCV can easily justify higher HCC incidence due to direct oncogenic effects of viral proteins or to chronic inflammation and fibrosis associated with non-cured viral infection [11].

Secondly, there are the effects of DAAs in the potential restoration of adaptive and innate immune cell populations after HCV elimination. It is well documented that chronic antigen stimulation, as it occurs during persistent infection with HCV, is associated with prolonged activation and impaired function of several immune cell populations, such as natural killer (NK) and virus-specific CD4+ and CD8+ T cells and Mucosal-Associated Invariant T (MAIT) cells. The first response is mediated by interferon (IFN)-mediated cell activation and specifically, through the induction of IFN-stimulated genes (ISGs). Although not all HCV infected patients show detectable induction of ISG genes, this is the first line of defense against viral infection [12]. Despite the expression of ISG genes, HCV can persist for decades. After DAA therapy a rapid downregulation of ISGs is achieved [13] even though the ISG expression normalization does not necessarily correlate with sustained viral response (SVR).

NK cells from patients with chronic HCV infection show high expression of several activating receptors NKp30, NKp44, NKp46, NKG2C, and NKG2D [14] but do not exert effector functions possibly due to diminished production of anti-viral cytokine production such as IFN-α and TNF-α. With DAAs there is a decrease in NK cell activation followed by a restoration of normal phenotype and function of this immune cell compartment [15]. A correlation between a decrease in NKG2D activator receptor expression in NK cells and higher risk of HCC early recurrence has been described [16]. Here, a rapid decrease in NKG2D receptor after DAA therapy in comparison to patients receiving the IFN-combined treatments was reported. NK cells not only represent the first line of defense against viral infection, they also have an anti-tumor effect by direct lysis of tumor cells [17]. 

During persistent HCV infection, CD4+ and CD8+ T and MAIT cells are usually recruited to the liver attempting to eradicate the infection. The continuous exposure to viral antigens leads to a decrease in cytotoxic T lymphocytes functions developing an “exhausted phenotype” [18] characterized by an increase in the expression of inhibitory molecules such as programmed-death (PD-1), cytotoxic T-Lymphocyte antigen 4 (CTLA-4) and CD244 with their correspondent ligands. Similarly, MAIT cells are permanently activated during chronic HCV infection [19] and show signs of exhaustion. While DAA therapy diminishes T cell recruitment to the liver and allow T cells recovering from their exhausted phenotype [20] to fully exert the cytotoxic activity [21], MAIT cells do not recover either in number or in functionality after successful HCV cure [22]. The potential role of liver MAIT cells in controlling HCC development is still unknown.

All things considered, the abrupt virus elimination seems to open a temporal immunosuppressive phase due to immune reconstitution. In this scenario the potential presence of tumor dormant micronodules might find the ideal conditions to grow and become apparent. Tumor mass dormancy is the result of the equilibrium between the proliferation and death of cancer cells. This equilibrium may be achieved through changes in angiogenesis and/or immune system [23]. The initial reports of the existence of dormant cells derive from breast cancer [24] and Schmidt-Kittler and colleagues specifically found epithelial-specific cytokeratin-positive cells cancer in the bone marrow of patients with breast cancer with or without metastasis [25]. The genomic comparative hybridization of cancer cells in the bone marrow from patients without metastasis revealed a significantly lower chromosomal aberrations rate in comparison to primary tumors or cancer cells from metastatic patients. Similar findings have also been reported in prostate cancer patients [26]. The hypothesis of the immune system reacting against malignant cells was proposed by Paul Ehrlich in 1909 [27]. However, the cancer immunosurveillance hypothesis was rejected due to the lack of appropriate experiments. In this study they identified small, inert lesions containing tumor cells that were kept at low numbers thanks to an active immune system. Not only is the immune system able to inhibit tumor growth, but it is also able to change cancer phenotype. The lower immunogenicity of cluster of tumor cells is supposed to favor these cells due to the constant interaction with immune cells and may potentially lead to tumor outgrowth [28]. Thus, the transitory immunosuppressive phase after DAA treatment in addition to the presence of tumor cells with more aggressive behavior may be the mechanism responsible for the rapid tumor growth in these patients. 

As a matter of fact, it has been shown that there is an increase in hepatitis B and herpes virus reactivations after DAA therapy [29,30].

Furthermore, HCV is known to interfere with several intracellular signaling pathways and cellular functions in host cells. For instance, HCV proteins increase lipid synthesis and reduce secretion of very low-density lipoproteins leading to liver steatosis [31]. HCV also interferes with insulin signaling [32] and viral RNA sequesters miR122 [33] to enhance its own stabilization and replication leading to a global de-repression of miR122 target genes. Since miR122 is crucial for the long-term liver homeostasis and its inhibition results in development of progressive liver disease and spontaneous HCC in animal models [34], a robust hypothesis is that miR122 inhibition during HCV infection is a main driver of liver disease and HCC development during viral infection. Therefore, all these effects might be reverted after HCV clearance by DAAs. If this seems to be true in the case of insulin resistance, which is improved after DAA treatment [35], this improvement has not been confirmed regarding serum lipid profile [36]. On the other hand, the impact of DAA therapy on miR122 cellular functional recovery is still largely unknown. 

After HCV elimination, not all the cellular signaling pathways previously disturbed are restored to normal levels. It is known that HCV infection may induce global epigenetic changes in infected hepatocytes. Interestingly, the epigenetic profile may be imprinted in subsequent cell divisions and maintained by an unknown epigenetic memory mechanism [37] even following HCV cure with DAAs. Accordingly, the development of HCC after SVR may be associated with a specific genetic signature in HCV-infected patients that persists even after antiviral cure [38,39].

Although HCV-associated epigenetic profile modification in hepatocytes is related to increased risk of HCC in cirrhosis, whether this prompts different results after DAA or IFN-combined treatments is something that deserves further investigation.

## 3. Time Association between DAA Treatment and HCC Recurrence

All these factors could explain, from a mechanistic point of view, the phenomena of ‘time association between DAA treatment and HCC recurrence’ reported by our group and the Italian group [40,41,42]. Several authors validated these data even though it was not reported in the conclusion of their manuscripts (Table 1 [40,41,43,44,45,46,47,48,49,50,51,52,53,54] adapted from [55]). In this regard, our original manuscript was focused on patients who achieved complete response (CR) without non-characterized nodules (no-ChN) to avoid an over-interpretation of the HCC recurrence rate. Cabbibo et al. [45] subsequently published a similar study on the same profile of patients and reported that the probability of HCC early recurrence in patients who had HCC previously cured remains high, despite HCV eradication by DAAs and concluded that the risk was comparable but not higher to that reported in the literature on DAA-untreated patients. However, according to the information reported in Figure 4 of that publication, the rate of recurrence ranges from 40% to > 60% within the first-year of ending DAA therapy. It is important to note that all patients included in that study were BCLC0/A patients who achieved CR and the time between CR to DAA initiation was not considered in the analysis. In that study [45], as well as in the Ogawa et al. [56] study, the authors demonstrated that the tumor size and prior-history of recurrence [45] and number of HCC nodules [56] were predictors of HCC recurrence. However, the time between CR and DAA initiation was not identified as a predictor in the Cabibbo cohort [45] though it was in the Ogawa study [56]. Interestingly enough, in the Cabbibo et al. cohort [45] the patients were divided according to the Barcelona Clinic Liver Cancer (BCLC) [57] staging but in the Ogawa cohort [56] patients were not stratified according to a no-staging system. Thus, this methodological bias could explain in part the discrepancy between both studies. In this regard, according to these data the strategy of ‘delaying 6–12 months the DAA initiation’ to avoid HCC recurrence is not supported. 

In addition, a recent study from Allaire et al. [58] analyzed the rate of recurrence and overall survival in patients treated with radiofrequency in all etiologies. They analyzed the HCV patients separately and evaluated the impact of SVR according to the regime received (DAA versus IFN). Once more, the results were not conclusive with HR for recurrence 0.93 (95% C I 0.25–3.50) and a HR 1.37 (95% CI 0.26–7.14) for OS in SVR-DAA in comparison with SVR-IFN. These results mean that the risk of developing HCC recurrence in patients who achieve SVR with DAA ranges between 0.25 times less and 3.5 times higher than those who achieve SVR with IFN (Supplementary Table S3 of Allaire et al. [58]). The same issue was observed in the risk of mortality analysis where the risk of death in patients who achieved DAA-SVR was between 0.26-fold lower and 7.14-fold higher [58]. This means that the risk of death by DAA is inconclusive in comparison to SVR-IFN. In addition, it could be up to 7-fold higher than the risk of death in SVR-IFN in the setting of patients with a history of HCC because the highest CI95 was 7.14. Indeed, these data reinforce the idea that the ‘host condition’ is disrupted in the setting of DAA exposure and has an impact on LC development regardless of the time between CR to DAA initiation. 

## 4. Presenting Non-Characterized Nodules before DAA Treatment and the Risk of Developing HCC

The multicenter cohort from Spain [59] supports the hypothesis that patients without previous HCC but presenting non-characterized nodules before DAA treatment have a higher risk of developing HCC than patients without. The incidence of de novo HCC in a cohort of 1123 cirrhotic patients was 3.73 HCC/100 patients-year (95% CI 2.96;4.70) but the relative risk was significantly increased in patients with non-ChN at baseline 2.83 (95% CI 1.55;5.16) versus absence of non-ChN. This clinical observation suggests that patients with this condition have a higher risk of developing de novo HCC than those who do not, but this does not mean that the risk is lower than for those who do not have non-characterized nodules before starting DAA [59]. In addition, this observation was also reported by Nahon et al. [60] (Supplementary table 14). They reported that 5 out the 12 patients (41.7%) who had screening procedures before starting treatment developed an HCC following DAA therapy. All of them presented non-characterized nodules before DAA treatment. Thus, these data also support the hypothesis raised by our group in 2016 [40] regarding the changes in the ‘dormant clones’ status in the context of DAA treatment. These cells are under immunological control but in the context of DAA treatment they can shift and escape control and develop de novo HCC or HCC recurrence. In this regard, the immunological disruption is also manifested by virus reactivation [29,30], thrombotic [61] or immunologic events such as angiogenesis (serum liver angiopoietin-2 and VEGF) [62], an imbalance of cytokine network, a reduction in CD8+ cellular density and a rapid downregulation of ISGs [63].

## 5. Serum Markers as Predictors of HCC Development

Another view of these phenomena is to consider the combination of factors such as the failure to achieve SVR, a favorable microenvironment for HCC evolution or epigenetic changes that are not modified despite having been cured of HCC. For the moment, however, it is not possible to characterize patients before starting DAA treatment and this hypothesis needs to be validated.

Prenner et al. [47] observed that of the 29 patients with HCC who did not achieve SVR, 27 (93%) presented active HCC at the start of DAA treatment. This observation led the authors to consider the presence of active HCC at DAA initiation as one of the factors related to a lower rate of SVR in comparison to those without active HCC at the time of starting DAA treatment. However, this observation was not validated in other cohorts (Table 2). Thus, the outcome of patients with a prior history of HCC differs, as expected, according to the activity of the HCC when DAA treatment is initiated. The evolution of patients, however, is heterogeneous despite similar tumor burden and/or tumor activity. This observation ties in with the hypothesis that some host conditions, such as angiogenic profile [64], could determine patient outcome. Unfortunately, we do not currently have clinical, radiological, or circulating markers to identify those patients with no or low-risk of developing HCC before DAA initiation where the benefit of DAA treatment would be unequivocally superior than the risk of developing an aggressive HCC. 

According to Faillaci et al. [64] the level of VEGF is increased during DAA-treatment and could act as a trigger in predisposed patients (severe fibrosis or splanchnic collateralization) who already show high activation of neo-angiogenesis pathways in cirrhotic tissue. In addition, in RNA extracted from tumor hepatic tissue, the angiopoietin-2 levels were significantly higher in patients with recurrent HCC (median fold change, 2.19) and de novo HCC (median fold change, 3.91) than in those with non-recurrent HCC (median fold change, 1.00; *p* = 0.005).

Finally, Ogawa [56,65] also evaluated the impact of AFP at the time of treatment completion and the results are also controversial. Thus, according to the current data, the impact of DAA on HCC patient outcome needs to be clarified and we need to wait until prospective studies or metanalysis with individual data are available. 

## 6. Impact of HCC Recurrence on the Overall Survival of Patients Treated with DAA

All the factors mentioned above could have a role in HCC recurrence but as pointed out by Cabbibo et al. [66] the aim of treating HCC and HCV is to improve the OS. In this regard, they evaluated the risk of death in HCV patients who achieved HCC CR after curative treatment but who did not receive HCV treatment to evaluate the natural history of this population. Interestingly enough, the risk of developing recurrence (21, 41.6, 61, and 64%) was higher than the risk of decompensation (10, 21.3, 36, and 44%) at 1, 2, 3, 4, and 5 years. According to our interpretation, which differs from the authors, these data support the fact that HCC recurrence risk drives the outcome of patients with a history of HCC. It is already known that almost all patients who develop HCC progression die due to cancer-related symptoms such as liver failure or cirrhosis complications. For this reason, indirect parameters could help the physician to assess if the death was related or not to the HCC. According to Cabbibo et al. [66] only 2% of the untreatable HCV patients received sorafenib and no patient received best supportive care (BSC) when they developed the first HCC recurrence. However, in our cohort [67] 16.7% of the 20 BCLC 0/1 HCV-DAA treated patients received BSC when developing HCC recurrence. This observation was also shared by Colombo et al. in a letter published last year [3] and reflected in the 1-year mortality rate in our cohort, and in Cabibbo’s et al. treated and untreated cohorts [45,68] (Table 2). 

## 7. The Minimum Information That the Studies Need to Report in Order to Disregard the Alarm

**Figure 1:**
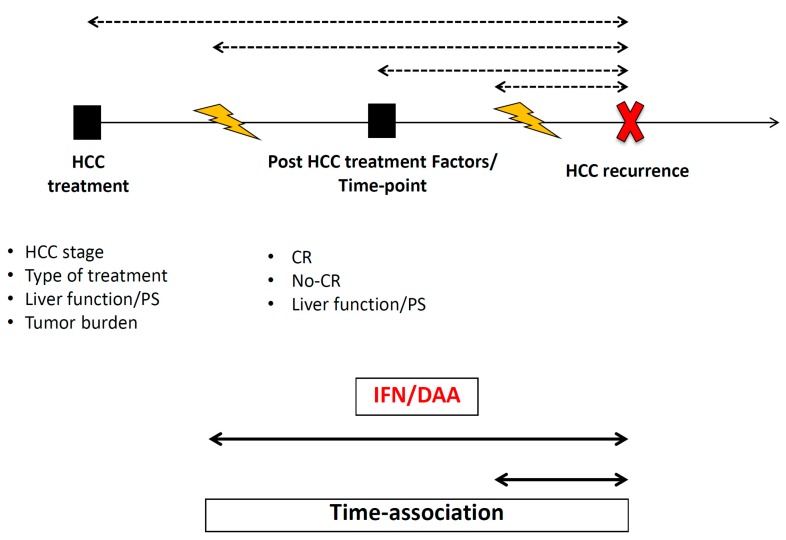
The minimum information that the studies need to report in order to disregard the alarm. The black squares represent the start point of the analysis and the note below each black square indicates the minimum information that the studies need to report at each point. The red X marks the event (HCC recurrence) and the yellow flash represents the start point of HCV treatment. The dotted lines reflect the different follow-ups according to the selected start point (at HCC treatment, at post-HCC treatment or at the time of starting HCV treatment regardless of the type of treatment). The continuous lines represent the time between starting HCV treatment and the date of HCC recurrence diagnosis.

**Figure 2:**
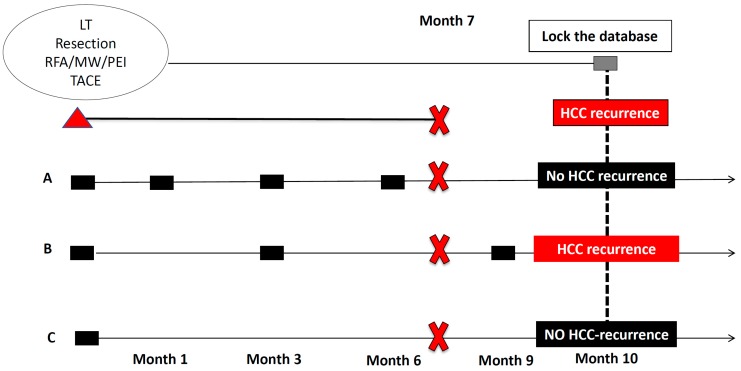
Impact of radiological evaluation on the rate of HCC recurrence reported. The red triangle represents the start point (the day of the treatment-LT, resection, RFA/MW/PEI or TACE-, the red cross represents the event (HCC recurrence) and the line between the two the time between treatment to HCC recurrence (7 months). The grey square represents the information inserted in the database at the day of the analysis (month 10). If this patient is evaluated in 3 different centers, which have a different radiological schedule to evaluate the radiological response, the information in the database will be different despite the patient being exactly the same. Centre A performs radiological evaluations at month 1, 3, and 6; Centre B at month-3 and 9; Centre C does not perform a defined radiological evaluation. However, all the centers have a database. Thus, if the patient develops a recurrence at month 7 and the database is locked at month-10, this patient will be considered a patient with HCC recurrence in center B because the information is in this database but no in centre A and B because the information is not in the database despite the fact that the event has already developed.

The minimum information that the studies need to report in order to disregard the alarm that we raised in 2016 is summarized in Figure 1. Briefly, studies need to report the starting point of the analysis, the evolutionary points used in the analysis to define the follow-up (treatment start date, post-HCC treatment date and the date of recurrence/progression), the characteristics of patients (stage, treatment received, activity status, liver function status, and PS), the original aim when the data collection was designed as well as the radiological/clinical and biochemical follow-up of patients. Indeed, the main concern regarding the discrepancies reported in the current data about this topic could be related to the radiological schedule used to evaluate the cohorts. Figure 2 describes the impact of radiological evaluation on the rate of HCC recurrence reported. 

## 8. Conclusions

The issue of HCC development after cure of hepatitis C is unresolved and the guidelines maintain the recommendation of treating HCV in patients with prior history of HCC until further information is available. 

If we consider that some authors suggest that the rate of SVR is lower in HCC patients than in non-HCC patients, the strategy of waiting >12 months after HCC treatment is still contradictory and 2 prospective cohorts from the same research group reported a higher 1-year rate of death in those patients who received DAA treatment. We encourage the reader to keep in mind the risks and benefits of treating individual patients using the same philosophy established by the guidelines but challenge them to consider an alternative approach to ‘until further information is available…’. If the patient has an HCC under complete response and the liver function is preserved, why do you need to treat them? 

## Figures and Tables

**Table 1:** Studies reporting analysis of hepatocellular carcinoma (HCC) recurrence after direct antiviral agents (DAA) treatment according to the rate of sustained viral response (SVR).

Authors, Year	Journal	Type of Publication	Type of Study	N	SVR (%)		
					ACTIVE HCC	HCC CR	No HCC
Reig M, 2016	J Hepatol	Original	Retrospective	58 (HCC CR)	--	97.5	--
Conti F, 2016 *	J Hepatol	Original	Retrospective	59 (HCC CR) 285 (No HCC)	--	89.8	91.6
The ANRS collaborative study group on HCC, 2016	J Hepatol	Original	Prospective	189 (HCC CR) ^1^ 13 (HCC CR) ^2^	--	91.9^1^ 100^2^	--
Ikeda K, 2017	Dig Dis Sci	Original	Retrospective	177 (HCC CR)	--	89.6	--
Cabibbo G, 2017	Aliment Pharmacol Ther	Original	Prospective	143 (HCC CR)	--	96	--
Beste LA, 2017	J Hepatol	Original	Retrospective	482 (Active HCC) 17 (No HCC)	74	--	91.1
Prenner SB, 2017	J Hepatol	Original	Retrospective	59 (Active HCC) 76 (HCC CR) 284 (No HCC)	57	97	88
Saberi B, 2017	Hepatology	Original	Retrospective	21 (Active HCC)	67	--	-
Adhoute X, 2018	Eur J Gastr Hepatol	Original	Retrospective	22 (HCC CR)	--	86	--
Hassany M, 2018	Eur J Gastr Hepatol	Original	Prospective	62 (HCC CR)	--	64.5	--
El Kassas M, 2018	J Viral Hepat	Original	Prospective	53 (HCC CR)	--	77.4	--
Abdelaziz AO, 2018	Eur J Gastr Hepatol	Original	Retrospective	45 (HCC CR) 44 (No HCC)	--	64.4	70.5
Sugiura A, 2018	J Viral Hepat	Original	Prospective	79 (HCC CR) 759 (No HCC)	--	87.3	95.5
Leo A, 2018	Dig Liver Dis	Original	Prospective	161 (HCC CR) 1766 (No HCC)	--	95	95.1

HCC: hepatocellular carcinoma; SVR: sustained virological response; CR: complete response. * SVR = 91% (344 patients). ^1^ HEPATHER, ^2^ CIRVIR.

**Table 2:** Characteristics of the studies reporting HCC recurrence after DAA treatment or no-treatment.

Author	Reig et al.	Conti el al./Renzulli et al.	Cabibbo et al.	Cabibbo et al.
**Publication**	Journal of Hepatology 2016	Journal of Hepatology 2016	Aliment Pharmacology Therapy 2017	Journal of Hepatology 2017
	Seminar Liver Disease 2017	European radiology 2017		
**Type of study**	Retrospective		Prospective	Retrospective
**HCC treatment**	RFA/PEI/ Resection/TACE			RFA/PEI/ Resection
**BCLC 0/A (%)**	97.4	98.3	100	100
**HCC status before DAA initiation**	CR	3 patients without CR	CR	CR
	without non-characterized nodules		without non-characterized nodules	
**HCV-status**	All			no-SVR
**n**	77	59	143	328
**Follow-up**	At least one radiological assessment			
**Median Follow-up (months)**	12.4 (IQR: 8.4–18.7)	12.4 (range 1.5–90.2)	8.7 months (range 3–19)	27 (range 3–95)
**HCC recurrence %**	31.2%	30.5%	20.3%	43.3%
**6-months**	NA		12%	
**12-months**			26.6%	21%
**18-months**			29.1%	
**24-months**				41%
**Death (%)**	6.5%	NA	4.2%	1^st^ year-3%

HCC: hepatocellular carcinoma; BCLC: Barcelona Clinic Liver Cancer; DAA direct antiviral agents; HCV hepatitis C virus; RFA: radiofrequency ablation; PEI: percutaneous ethanol injection; TACE transarterial chemoembolization
